# Path-coefficient and correlation analysis in Bambara groundnut (*Vigna subterranea* [L.] Verdc.) accessions over environments

**DOI:** 10.1038/s41598-021-03692-z

**Published:** 2022-01-07

**Authors:** Md Mahmudul Hasan Khan, Mohd Y. Rafii, Shairul Izan Ramlee, Mashitah Jusoh, Md Al Mamun

**Affiliations:** 1 Laboratory of Climate-Smart Food Crop Production, Institute of Tropical Agriculture and Food Security (ITAFoS), Universiti Putra Malaysia (UPM), UPM Serdang, 43400 Selangor, Malaysia; 2 Department of Crop Science, Faculty of Agriculture, Universiti Putra Malaysia (UPM), UPM Serdang, 43400 Selangor, Malaysia; 3 Bangladesh Agricultural Research Institute (BARI), Gazipur, 1701 Bangladesh; 4 Bangladesh Jute Research Institute (BJRI), Dhaka, Bangladesh

**Keywords:** Plant breeding, Agricultural genetics

## Abstract

In a breeding program, studies of genotypic and phenotypic relationships among agricultural crop traits are useful to design, evaluate, and develop selection criteria for desirable traits. Using path coefficient analysis, the present study was executed to estimate the phenotypic, genotypic, and environmental correlation coefficients between yield and yield-related traits and to determine the direct and indirect effects of yield-related traits on yield per plant. A total of 30 genotypes of *Vigna subterranea* were studied under tropical conditions at two sites over two planting seasons (considered as four environments). The experiment at each site used a randomized complete block design with three replicates. Data were collected on vegetative and yield component attributes. Based on analysis of variance, pooled results showed that there were positive and highly significant differences (p ≤ 0.01) among the 30 genotypes for all attributes studied. Highly significant and positive strong correlation at phenotypic level was observed for dry seed weight (0.856), hundred seed weight (0.754), fresh pod weight (0.789), and total pod weight (0.626) with yield in kg per hectare, while moderate positive correlations were observed for harvest cut (0.360) and days to maturity (0.356). However, a perfect positive correlation was observed for the dry weight of pods with seed yield. In contrast, days to 50% flowering (− 0.350) showed a negative significant relationship with yield per hectare. The dried pod weight attribute (1.00) had a high positive direct effect on yield. Fresh pod weight had the greatest indirect effect on yield per hectare, followed by the number of total pods by dry pod weight. As a result, dry pod weight, hundred seed weight, number of total pods, and fresh pod weight could be used as selection criteria to improve the seed yield of Bambara groundnut (*Vigna subterranea*).

## Introduction

Bambara groundnut (*Vigna subterranea* [L.] Verdc.) is a grain legume grown in Sub-Saharan Africa (SSA) as a source of food and nourishment^1^. After groundnut and cowpea, Bambara groundnut is Africa's third most significant legume. According to Khan et al.^2^, it is highly adaptable to low-input farming systems and is one of the legumes preferred by various resource-limited farmers. The bambara nut is a nutrient-dense legume that is commonly referred to as a "complete food." Dried Bambara Seeds have a carbohydrate content of 64.4%, a protein content of 23.6%, a oil content of 6.5%, and a fiber content of 5.5%^3^. Because of its high ranking among legumes, the world's population's exponential growth rate has forced an increase in current production of this essential legume. As a result, scientists have investigated a variety of approaches to cope with this persistent challenge. Previously, various scientists successfully carried out study on Bambara groundnut, such as morphological variation in response to photoperiod^4,5^, planting date (time)^6,7^, moisture deficit^6,7^, temperature on leaf development^8^, and reaction to soil moisture^9^. Although Bambara groundnut cultivars naturally reacted differently, this provides a fantastic potential for the creation of Bambara groundnut variants.

According to a Khan et al.^10^ assessment, yields are now uncertain and low due to a lack of established cultivars and farmers' continuous usage of native landraces. However, multiple studies have found that this crop has the potential to produce higher yield of 1049 kg/ha^11^, 1180 kg/ha^12^, and 2445–5267 kg/ha^13^. The study of genetic inconsistency is important for plant species in terms of disease and insect resistance, as well as for plant breeders in addition to increasing breeding success of economically significant traits such as yield^14^. Plants with desirable features survive better than inferior ones in strong climate variations, therefore sufficient genetic diversity promotes plant sustainability.Morphological features have been employed in crop development to create stable breeding lines with high yield in a wide range of environmental conditions, such as chickpea^15^, groundnut^16,17^, and Caribbean bean landraces^18^.

Morphological indicators were employed to infer evolutionary relationships between the lines. Such findings demonstrate that, despite the reality that morphological indicators might be influenced by the environment, their application is imperative, particularly in neglected crops^14^.

Yield is a complicated variable that is influenced by a variety of factors such as polygenes, environment, and genetic heterogeneity^19^. Selection for higher pod yield should not be focused just on yield due to its complexity and interaction with other yield-enhancing traits. As a result, various yield-enhancing characteristics should be considered. Path coefficient analysis is a valid statistical method for dividing correlation coefficients into direct and indirect effects. It evaluates the connectivity of several yield-relevant characteristics.

Perhaps it has a direct effect on yield or follows a different path for total influence, allowing the contribution of each trait to yield to be identified. This strategy is used as a selection aid for genetic as well as yield improvement in plant breeding programs. The characteristics of the yield components do not occur independently; rather, they are interconnected and result in pod yield in Bambara groundnut. Path coefficient analysis examines the effects of predictor parameters as first and second-order component variables on a dependent variable such as yield^19^.

The traits such as plant height, days to 50% flowering, days to maturity, number of branches, the total number of pods, biomass fresh weight, dry pod weight, dry seed weight, hundred seed weight, and harvest index are all-important yield components that need to be considered before starting an effective breeding program^14,20^. Path analysis, according to Anwar et al.^21^, may be used to investigate the measurable effect of direct or indirect influences on yield by one or more parameters. Path coefficient analysis is widely used by researchers in chilli^19^, wheat^22^, canola^23^, and cowpea^24^ to clearly explain the relationships among yield-enhancing factors. Crop yield enhancement may be achievable by breeding its contributing components if the yield-enhancing traits are highly heritable and have a favorable association with ultimate yield. The use of correlation tests (genotype, phenotype and environment), the interaction of two or more traits is crucial in understanding how an advance in one trait can cause concurrent changes in other traits^25^. To produce high-yielding varieties, it is necessary to study the heritable (genetic) differences for plant growth, yield, and yield component traits that are influenced by genetic and environmental factors^26^.

In the literature, there has been insufficient or no research on correlation-based path analysis and trait association, inheritance, and genetic progress in quantifiable attributes of V. subterranea in tropical conditions. Because the correlation coefficient simply measures the relationship between two traits, it does not show the relative value of each attribute. The current study employed path coefficient analysis approaches to evaluate the relationships and effect of significant yield contributing components on the yield of Bambara groundnut. The purpose of this study was to identify the degree and nature of the correlation between pod yield and contributing traits. These results will subsequently be used as selection criteria for increasing Bambara groundnut production and for future research in tropical and subtropical environments.

## Materials and methods

### Plant materials

The research work was conducted under the Institute of Tropical Agriculture and Food Security (ITAFoS), University Putra Malaysia (UPM), Malaysia. A set of 30 accessions of *V. subterranea* was used in this study taken from the GenBank of ITAFoS, UPM. In the beginning, fifteen collected accessions were carried out the formal identification and investigation by Md Mahmudul Hasan Khan^11,12^ under the direct supervision of Prof. Dr. Mohd. Rafii Yusop, Director, ITAFoS, UPM, Malaysia with follows the proper national and international strategies. During the evaluation, to select the genotypes selection was made from each selfed generation of S_0_ to S_5_ based on high yield. From the 15 evaluated landraces of generation S_0_ we selected 150 individual plants based on the maximum number of pods and yield per plant and undergoes subsequent selfing and selection considered as S_1_. These seeds were grown for selfing and advancing the next generation and selected 44 best performing lines. Likewise, after the execution of two rounds (S_2_ and S_3_) of selfing and selection, we planted the seeds of S_3_ and S_4_ generation together for comparative and inbreeding depression study. Moreover, molecular characterization (https://doi.org/10.1038/s41598-021-93867-5) was also executed using the 44 accessions of S_4_ selfed generation. However, the seeds of all selected promising lines are deposited at GenBank, ITAFoS, UPM. Finally, we selected the 30 bests performing lines of *V. subterranea* from the 44 accessions of forth selfed (S_4_) generation based on the high yield and homogeneity of traits and considered them to be the S5 selfed generation. In terms of plant guidelines, it has complied with relevant institutional, national, and international guidelines and legislation**.** We collected the plant seeds or specimens with the proper permission of the institution's authority by following the national and international strategies as well as deposited them in GenBank, ITAFoS, UPM. We also took appropriate permission from the farm or field owner during specimens’ collection and experimentation. We provide confirmation that during collection and execution of the experiment authors have complied with the IUCN Statement on Research Involving Species at Risk of Extinction and the Convention on the Trade in Endangered Species of Wild Fauna and Flora. The name and ID of each accession are listed in Table 1.

**Table 1:** The thirty selected Bambara groundnut accession used in this study.

Genotype	ID	Genotype	ID	Genotype	ID
Maik12-18	S5G1	GiiwP12-18	S5G11	GiiwP9-18	S5G21
MaikP3-18	S5G2	ExSokP4-18	S5G12	GiiwP11-18	S5G22
MaikP6-18	S5G3	KarP10-18	S5G13	KarP8-18	S5G23
BdilaP5-18	S5G4	MaikP11-18	S5G14	DunP6-18	S5G24
JataP1-18	S5G5	MaibP8-18	S5G15	GiiwP1-18	S5G25
DunP9-18	S5G6	MaibP6-18	S5G16	KataP5-18	S5G26
CancP3-18	S5G7	KataP8-18	S5G17	KarP9-18	S5G27
RokP1-18	S5G8	DunP2-18	S5G18	DunP8-18	S5G28
ExSokP5-18	S5G9	CancP2-18	S5G19	RokP9-18	S5G29
ExSokP3-18	S5G10	BdilaP8-18	S5G20	JataP3-18	S5G30

Maik = Maikai; Bdila = Bildillali; Jata = Jatau; Dun = Duna; Canc = Cancaraki; Rok = Roko; Giiw = Giiwa; Kar = Karu; Maib = Maibergo; Kata = Katawa; Exsoko = Exsokoto.

### Environment and location

Field trials were conducted recurrently at two sites in two cropping seasons (2020 and 2021) in Malaysia. The environments (combination of seasons and location) spanned a considerable range of conditions in terms of temperature (warm vs. temperate climate), rainfall (heavy rain vs. supplemental irrigation), soil structure, soil pH, and management practices (researcher's field vs. farmer's field). Details of environmental conditions were presented in Table 2. The soil properties of the experimental site are presented in Table 3.

**Table 2:** Environmental description of the experimental site.

Code	Season	Latitude	Longitude	Altitude	Av Temp	Av Hum (%)	Rainfall (mm)	Year
FTM (ENV 1)	Main	2.990935	101.7138	61.0 m	23.14–29.88 °C	83.2	188.6	2020
FTO (ENV 2)	Off	2.990935	101.7138	61.0 m	24.22–30.72 °C	82.6	198.4	2021
FFM (ENV 3)	Main	2.983092	101.7152	54.0 m	23.14–29.88 °C	83.2	188.6	2020
FFO (ENV 4)	Off	2.983092	101.7152	54.0 m	24.22–30.72 °C	82.6	198.4	2021

FTM = Field ten main season; FTO = Field ten off season, FFM = Field fifteen main season; FFO = Field fifteen off season; ENV. = Environment; Main season = May- September; Off season = November–March; Av. Temp. = Average temperature; Av. Hum. = Average humidity. Sources: https://en.climate-data.org/asia/malaysia/selangor/mardi-serdang-971613/#climate-table.

**Table 3:** Characterization of soil properties of the experimental region.

Determination	Location	
	Field fifteen (FF)	Field ten (FT)
Physical analysis	Value	
Sand (%)	40	5.8
Silt (%)	26.82	51.19
Clay (%)	33.74	42.99
Textural classes (USDA)	Clay loam	Silty clay

Chemical analysis	Value	
pH	6.6–7.5	5.0–5.59
Organic matter (%)	1.97	10.32
Total nitrogen (%)	0.16	0.41
Available phosphorus (mg kg^-1^)	10.6	59.2
Available potassium (mg kg^-1^)	120.6	306.4
Khan et al.^12^		

### Design and layout of experiment and plant husbandry

This current investigation was set up in a Randomised Complete Block Design (RCBD) with three replications in each environment. Each experimental unit consisted of two rows of 1.6 m × 0.80 m each. According to Khan et al.^12^, the spacing was maintained as 30 cm for a plant to plant, row to row 50 cm, plot to plot 1.5 m, and between two replications 2.0 m. Moreover, the plant population density was maintained as 6 plants m^-2^. During the growing season, prescribed agronomic operations such as field planning, land clearing, weeding, irrigation, and fertilization were carried out. The prescribed fertiliser rates (100% N = 45 kg N/ha, 100% P = 54 kg P_2_O_5_/ha, 100% K = 45 kg K_2_O/ha) were applied at final tillage, with 70% N added five weeks after planting^11,27^. The field was mechanically ploughed at the study sites following the usual cultural traditions of the local farmers. Regular hand weeding as well as pest and disease control measures were carried out whenever needed.

### Data collection

For data collection, a total of twelve quantitative features were taken into account based on Bambara groundnut descriptions and descriptors from IPGRI, IITA, and BAMNET^28^. For each trait, data were collected on five randomly selected plants, from each genotype and replication. The collection of quantitative data included as (1) days to 50% flowering = D50%F (d), (2) days to maturity = DTM (d), (3) plant height = PH (cm), (4) number of branches per plant = NB, (5) biomass fresh weight per plant = BFW (g), (6) total number of pods per plant = TNP, (7) fresh pod weight per plant = FPW (g), (8) dry seed weight per plant = DSW (g), (9) hundred seed weight per plant = HSW (g), (10) harvest index = HI (%), (11) dry pods weight per plant = DPW (g), and (12) pod yield (kg per hectare). Of the quantitative traits studied, days to 50% flowering, days to maturity, plant height, and number of branches per plant were verified in the field and the remaining traits were measured in the laboratory after harvest.

### Statistical analysis

The SAS 9.4 (Statistical Analysis System) was used to calculate genotypic and phenotypic correlation coefficients (SAS Institute Inc., Cary, NC, USA). According to Kashiani and Saleh^29^, the genotypic and phenotypic correlations of several traits with yield per hectare were studied. Using Wright's^30^ method and path coefficient analysis, the genotypic correlations were then partitioned into components of direct and indirect effects. Misangu et al.^31^, Usman et al.^19^, and Oladosu et al.^32^ developed a technique for finding path coefficients that used a matrix notation to illustrate the relationships between correlations and path coefficients. Based on two-level relationships, the traits studied were then divided into first- and second-order components. The traits such as days to 50% flowering (D50%F), days to maturity (DTM), plant height (PH), number of branches per plant (NB), and biomass fresh weight (BFW) were considered as first-order components, while the second-order components are the total number of pods per plant (TNP), fresh pod weight per plant (FPW), dry pod weight per plant (DPW), dry seed weight per plant (DSW), hundred seed weight (HSW) and harvest index (HI). The cause and effect correlations between the two components were obtained using simultaneous equations in matrix notation. All the related equations can be found as “Supplementary Table S1” online.

## Result and discussion

### Sources of variation analysis

Table 4 displays the combined analysis of variance results for all genotypes across all locations for the traits of vegetative, yield, and yield components. As shown in Table 4, significant variation was observed among environments (E), genotypes (G), and G × E interaction. Highly significant differences (p ≤ 0.01) were detected between environment, genotype and genotype-environment interaction for all the variables studied. The extent of the significant differences observed implies that there is a considerable degree of genetic variation among the genotypes evaluated. The coefficient of variation (CV %) for yield and yield-related components varied from 5.43% (DTM) to 26.24% (HSW), showing that there is substantial heterogeneity across the traits evaluated. Any breeding material with high genetic variation has a greater chance of obtaining desirable traits and can be effective in heterosis breeding^33^. The differences in genotypes might be attributed to their genetic histories and origins from a range of sources. In this regard, several types of research on phenotypic diversity among Bambara groundnut genotypes have been published viz. Suneetha^34^ identified a roughly similar range of variance across the 29 groundnut cultivars. Environmental influences on yield components had a considerable impact for all genotypes, according to Masindeni^20^. A significant difference (p ≤ 0.01 and 0.05, respectively) was observed between genotypes and genotype by environment interaction and these results were in agreement with the findings of Pranesh et al.^35^, Shegro et al.^36^, Jonah et al.^37^ and Naik^38^ in Bambara groundnut. Bambara groundnut showed considerable variation in morphological and yield characters^11,12,14,39^. Ambrose^40^ accepted yield as a source of information, and ANOVA revealed that location accounted for 97.22%, while genotypes and genotype by environment interaction (G x E) accounted for 0.58% and 0.61%, respectively.

**Table 4:** The mean squares and significant level of vegetative, yield, and its related traits revealed by ANOVA.

Source of variation	Reps (Environment)	Environments (E)	Genotypes (G)	G × E	Error	CV (%)	St. Dev
df	8	3	29	87	232		
D50%F	170.93**	606.94**	124.08**	32.67**	5.11	14.09	5.49
DTM	186.58**	177.29**	265.12**	74.60**	10.46	5.43	7.2
PH	69.60**	663.21**	24.28**	13.63**	4.48	13.24	3.91
NB	122.61**	1668.60**	109.37**	51.20**	17.58	18.1	7.02
BFW	14,799.31**	738,639.89**	103,871.17**	3875.17**	420.23	27.96	126.9
TNP	1042.51**	831.01**	220.44**	71.03**	19.44	10.41	8.82
FPW	9959.17**	428,488.77**	19,117.35**	6651.85**	256.82	13.32	84.41
DPW	4697.92**	191,292.95**	6228.69**	170.41*	127.54	11.98	47.4
DSW	1918.50**	75,582.15**	4193.77**	420.84**	275.9	11.77	35.96
HSW	1886.90**	216,273.59**	1945.19**	204.52**	130.21	26.24	46.26
HI	11.65**	424.48**	403.52**	4.28**	2.16	10.01	6.23
YLD	166,448.32**	6,423,330.79**	220,685.25**	6038.15*	4519.1	11.98	282.13

df = degrees of freedom, St. Dev = standard deviation, CV = coefficient of variation, * = significant (p ≤ 0.05),** = highly significant (p ≤ 0.05), D50%F = days to 50% flowering, DTM = days to maturiity, PH = plant height (cm), NB = number of branches , BFW = biomass fresh weight (g), TNP = total number of pods, FWP = fresh pods weight (g), DPW = dry pods weight (g), DSW = dry seeds weight (g), HSW = hundred seed weight (g), HI = harvest index, and YLD = yield kg/ha.

### Overall mean performance

The overall mean and comparison of each evaluated vegetative trait, yield, and yield component in this study are presented in Table 5. In this study, all of the evaluated traits exhibited considerable variation across the environments. Within the row values with different letters indicate that the traits performed differently in the environments tested, at least significant difference (LSD) = 0.05. Days to 50% flowering were recorded at 42 (d) for Ladang fifteen (LFO), which was greater than the other three environments, which averaged 38 (d). In the environment LTO, most of the genotypes took less time to maturity (130 days) which was statistically different from the other environments. However, with an average of 132 days, the LTM and LFO environments (133 days) had the largest number of days to maturity. Plant height (31 cm) was comparable in both seasons at location Ladang fifteen, but not statistically comperable in both seasons at location Ladang ten. The average number of branches per plant was 38, and there was a significant variation across the four environments. The off-season performance for biomass fresh weight at both locations was statistically similar, with a mean of 453.85 g, while the two major seasons had different values, such as 373.30 g for LTM and 583.87 g for LFM. The average number of pods per plant was 84, with the environment LFO accounting for the greatest number of pods (88), followed by LTM (85) which is statistically similar to LTO (84) but not LFM. The trait fresh pod weight (g) differed significantly; the highest value was recorded for the environment LFO (718.12 g) with an average of 633.61 g, and the lowest was 565.89 g for the main season of location Ladang ten. The yield was determined using the parameters dry pods weight per plant, and we identified a perfect positive correlation between these two traits.

**Table 5:** Overall mean performance of vegetative, yield, and yield component traits of 30 *V. subterranea* genotypes across the environments.

Traits	Location					
	LTM	LTO	LFM	LFO	Mean	LSD
D50%F	37.02c ± 0.50	36.86c ± 0.47	39.58b ± 0.44	42.38a ± 0.68	38.96	0.66
DTM	133.86a ± 0.72	130.83c ± 0.64	132.60b ± 0.78	133.73a ± 0.85	132.75	0.95
PH	29.54b ± 0.23	25.64c ± 0.32	31.60a ± 0.41	31.19a ± 0.34	29.49	0.62
NB	38.00c ± 0.37	43.59a ± 0.70	33.33d ± 0.76	40.26b ± 0.61	38.79	1.23
BFW	373.30c ± 9.57	430.31b ± 12.85	583.87a ± 5.44	427.90b ± 12.60	453.85	6.02
TNP	85.08b ± 0.78	84.95b ± 1.07	80.64c ± 0.85	88.02a ± 0.82	84.67	1.29
FPW	565.89d ± 3.45	659.96b ± 9.84	590.48c ± 4.26	718.12a ± 5.66	633.61	4.7
DPW	373.89c ± 2.42	381.80b ± 3.15	364.52d ± 2.29	462.05a ± 3.44	395.57	3.31
DSW	296.38b ± 2.36	296.3b ± 3.0	281.6c ± 2.36	347.69a ± 3.09	305.49	4.87
HSW	238.68a ± 2.46	133.43d ± 1.47	141.18c ± 1.02	192.01b ± 2.38	176.32	3.35
HI	60.60c ± 0.63	61.6b ± 0.71	61.34b ± 0.57	65.44a ± 0.61	62.24	0.43
YLD	2225.55c ± 14.40	2272.66b ± 18.73	2169.82d ± 13.60	2750.33a ± 20.64	2354.59	19.74

LTM = Ladang ten (main season), LTO = Ladang ten (off season), LFM = Ladang fifteen (main season), LFO = Ladang fifteen (off season), LSD = least significant difference (p = 0.05). Within a row, values with different letters indicate that the traits performed differently in the environments evaluated with LSD test at p > 0.05. D50%F = days to 50% flowering, DTM = days to maturiity, PH = plant height (cm), NB = number of branches , BFW = biomass fresh weight (g), TNP = total number of pods, FWP = fresh pods weight (g), DPW = dry pods weight (g), DSW = dry seeds weight (g), HSW = hundred seed weight (g), HI = harvest index, and YLD = yield kg/ha.

For the environment, the average dry pod weight was 395.57 g, with LFO having the highest dry pod weight of 462.05 g, followed by LTO (380.81 g) and LTM (373.89 g). Location Ladang ten had similar dry seed weight (296 g) values in both seasons, however, location Ladang fifteen had greater dry seed weight (347.69 g) with an off-season mean performance of 305.49 g. The recorded hundred seed weight was higher (238.68 g) in the main season of Ladang ten but the lowest was noted as 133.43 g for the off-season of the same location with a mean of 176.32 g. However, the performance of a hundred seed weight (g) in two seasons at Ladang fifteen was statistically not comparable. The environment LFO had the highest harvest index value (65%), while the Ladang ten had the lowest (60%). The yield was highly influenced by environmental factors however, maximum yield was observed in the off-season of location Ladang fifteen (LFO) as 2750 kg/ha, followed by off-season of Ladang ten (2272 kg/ha) with an average of 2354 kg/ha. We discovered that the off-season in both locations produced a higher yield than the main seasons. This is due to the effect of the environment on yield and its contributing factors. According to the meteorological data, off-season rainfall is higher than during the growing season, and sufficient moisture in the soil accelerates nutrient uptake by plants, enhancing plant growth and development as well as yield. Moreover, the experimental plot is irrigated by a well-arranged irrigation system to manage the soil moisture as well as experimental field condition. However the variation in yield performance is related to the seasonal imbalance of the environmental component is validated by Masindeni^20^, who studied Bambara groundnut in six different locations and his statemen is supported our findings. Ambrose^40^ demonstrated a comparable trend of variation in yield across two seasons and two locations using 49 groundnut accessions.

### Correlation analysis

The correlation coefficients (genotypic and phenotypic) for the vegetative characteristics, pod yield, and yield contributing factors evaluated in this study are shown in Table 6. The SAS program provides r-values and assesses their significance. The results demonstrated a significant positive relationship between yield and all parameters, with the exception of biomass fresh weight and days to 50% flowering. The yield characteristic does not express independently; rather, it exists as a result of an interaction with other factors, resulting in a complex interaction that ultimately affects yield. This interaction or relationship might be positive or negative. The r-value for Pearson's correlation coefficient aids in the discovery of a relationship between two independent variables, despite the fact that it does not indicate the degree of the association. Ratner^41^ describes a widely used approach for analyzing correlation coefficients. The correlation values for genotypic characters varied from (-) 0.030 (FPW vs. BFW) to 1.00 (DPW vs. YLD), whereas the correlation coefficients for phenotypic characters ranged from 0.029 (D50%F vs. BFW) to 1.00 (DPW vs. YLD). This shows that in most cases, the genotypic level is more closely related to the associated phenotypic level. Total number of pods (r = 0.62), fresh pod weight (g) (r = 0.78), dry seed weight (g) (r = 0.85), and hundred seed weight (g) (r = 0.75) all had a strong, positive, and highly significant correlation with yield. A perfect positive significant correlation (r = 1.00) was observed between dry pod weight and yield, whereas a moderate positive correlation was observed with the traits of days to maturity (r = 0.35) and harvest index (r = 0.36). It is preferable to select these four traits since they all contribute equally to seed yield improvement. Dry pod weight (g) per plant may show an important contribution because of its direct positive and perfect effect on yield kg per hectare. However, Table 7 displayed the environmental correlation of vegetative, yield, and yield component traits of 30 *V. subterranea* genotypes across the environment. We noticed that majority of the traits had a non-significant association with each other and with yield in terms of environmental correlation. The fresh pod weight (g) (r = 0.84) and dry pods weight (g) (r = 1.00) had strong and perfect correlation with yield. A week positively significant correlation was identified between the characteristics total number of pods vs. yield (r = 0.23), dry seeds weight vs. yield (r = 0.25), and hundred seeds weight vs. yield (r = 0.24), while a moderately meaningful correlation was found between harvest index vs. yield (r = 0.35) (Table 4).

**Table 6:** Assessment of phenotypic correlation (above diagonal) and genotypic correlation (bellow diagonal) among 12 characters of *V. subterranea* genotypes.

Variable	D50F	DTM	PH	NB	BFW	TNP	FPW	DPW	DSW	HSW	HI	Yld
D50F	1	0.388**	− 0.241*	− 0.215*	0.029 ns	− 0.475**	− 0.506**	− 0.544**	− 0.551**	− 0.432**	− 0.125 ns	− 0.544**
DTM	0.214*	1	− 0.094 ns	0.274**	− 0.079 ns	0.151 ns	0.313**	0.395**	0.434**	0.499**	0.168 ns	0.395**
PH	− 0.038 ns	− 0.034 ns	1	0.815**	0.332**	0.183 ns	0.127 ns	− 0.048 ns	0.058 ns	0.268*	− 0.320**	− 0.048 ns
NB	0.393**	− 0.036 ns	0.299*	1	0.414**	0.264*	0.236*	0.168 ns	0.085 ns	0.206 ns	− 0.355**	0.168 ns
BFW	0.060 ns	− 0.076 ns	0.269*	0.172 ns	1	0.080 ns	− 0.037 ns	− 0.162 ns	− 0.214*	− 0.252*	− 0.972**	− 0.162 ns
TNP	− 0.201*	0.041 ns	0.156 ns	0.341**	0.091 ns	1	0.825**	0.790**	0.735**	0.696**	0.058 ns	0.790**
FPW	− 0.387**	0.223*	0.086 ns	0.075 ns	− 0.030 ns	0.701**	1	0.834**	0.870**	0.766**	0.183 ns	0.834**
DPW	− 0.360**	0.366**	0.042 ns	0.095 ns	− 0.141 ns	0.636**	0.789**	1	0.986**	0.894**	0.364**	1.000**
DSW	− 0.315*	0.313*	0.116 ns	0.176 ns	− 0.169 ns	0.567**	0.756**	0.856**	1	0.944**	0.408**	0.986**
HSW	− 0.283*	0.404**	0.126 ns	0.096 ns	− 0.212*	0.511**	0.657**	0.754**	0.799**	1	0.412**	0.894**
HI	− 0.075 ns	0.137 ns	− 0.235*	− 0.049 ns	− 0.958**	0.069 ns	0.187 ns	0.360**	0.363**	0.363**	1	0.364**
Yld	− 0.350**	0.356**	0.042 ns	0.095 ns	− 0.141 ns	0.626**	0.789**	1.000**	0.856**	0.754**	0.360**	1

* = Significant (p ≤ 0.05),** = Highly significant (p ≤ 0.05), ns = non significant (p > 0.05), D50%F = days to 50% flowering, DTM = days to maturiity, PH = plant height (cm), NB = Number of branches , BFW = biomass fresh weight (g), TNP = total number of pods, FWP = fresh pods weight (g), DPW = dry pods weight (g), DSW = dry seeds weight (g), HSW = hundred seed weight (g), HI = harvest index, and YLD = yield kg/ha.

**Table 7:** Estimation of environmental correlation among 12 characters of *V. subterranea* genotypes.

Variable	D50F	DTM	PH	NB	BFW	TNP	FPW	DPW	DSW	HSW	HI
D50F	1										
DTM	− 0.024 ns	1									
PH	− 0.113 ns	− 0.092 ns	1								
NB	0.186 ns	0.185 ns	− 0.138 ns	1							
BFW	− 0.108 ns	0.104 ns	0.013 ns	− 0.052 ns	1						
TNP	− 0.122 ns	0.125 ns	0.020 ns	0.131 ns	− 0.091 ns	1					
FPW	− 0.104 ns	− 0.031 ns	− 0.008 ns	− 0.094 ns	0.009 ns	0.216*	1				
DPW	− 0.204 ns	0.014 ns	− 0.028 ns	− 0.142 ns	− 0.048 ns	0.237*	0.849**	1			
DSW	− 0.076 ns	− 0.004 ns	0.092 ns	0.110 ns	− 0.005 ns	− 0.006 ns	0.124 ns	0.258*	1		
HSW	− 0.014 ns	0.149 ns	− 0.253*	0.175 ns	− 0.010 ns	0.096 ns	0.188 ns	0.246*	0.360**	1	
HI	0.077 ns	− 0.086 ns	− 0.101 ns	0.076 ns	− 0.821**	0.201 ns	0.322**	0.355**	0.077 ns	0.135 ns	1
Yld	− 0.204 ns	0.014 ns	− 0.029 ns	− 0.142 ns	− 0.048 ns	0.237*	0.849**	1.000**	0.258*	0.246*	0.355**

* = Significant (p ≤ 0.05),** = Highly significant (p ≤ 0.01), ns = non significant (p > 0.05), D50%F = days to 50% flowering, DTM = days to maturiity, PH = plant height (cm), NB = Number of branches , BFW = biomass fresh weight (g), TNP = total number of pods, FWP = fresh pods weight (g), DPW = dry pods weight (g), DSW = dry seeds weight (g), HSW = hundred seed weight (g), HI = harvest index, and YLD = yield kg/ha.

Misangu et al.^31^, Oyiga and Uguru^42^, and Maunde et al.^43^ found a significant positive association and contributions of total pod number per plant and hundred seed weight. Dry pod weight per plant and number of pods per plant both contributed positively to yield in this study. Makanda et al.^44^, Alake et al.^45^, and Unigwe et al.^46^ provided clear evidence in Bambara groundnut in support of these conclusions. According to Ofori^47^, and Iqbal et al.^48^, days to 50% flowering and number of pods per plant are important traits in legumes, hence these traits should be prioritized for increasing seed yield in Bambara groundnut. Due to higher component compensation and uncertain climate circumstances, there was a strong positive association between hundred seed weight and yield with a minor contribution to yield (0.0357), which contradicted earlier findings by Karikari and Tabore^49^ and Misangu et al.^31^. However, our findings corresponded with those of Maunde et al.^43^, who found a direct influence of hundred seed weight on yield (0.0883). In our study, biomass fresh weight and days to 50% flowering had a negative relationship with yield, which is supported by Wigglesworth^50^, the negative association among the vegetative components could result from competition for ambient resources such as nutrients, light, moisture, genetic properties, and so on (linkage and pleiotropy). Maunde et al.^43^, Ofori^47^, Mogale^51^, Evangeline^52^, and Jonah^53^ in Bambara groundnut, Kumari and Sasidharan^54^, in groundnut, and Manggoel et al.^24^ in cowpea found comparable phenotypic and genotypic relationships between the total number of pods per plant and final yield.

#### Direct and indirect effects of vegetative traits on pod yield

**Figure 1:**
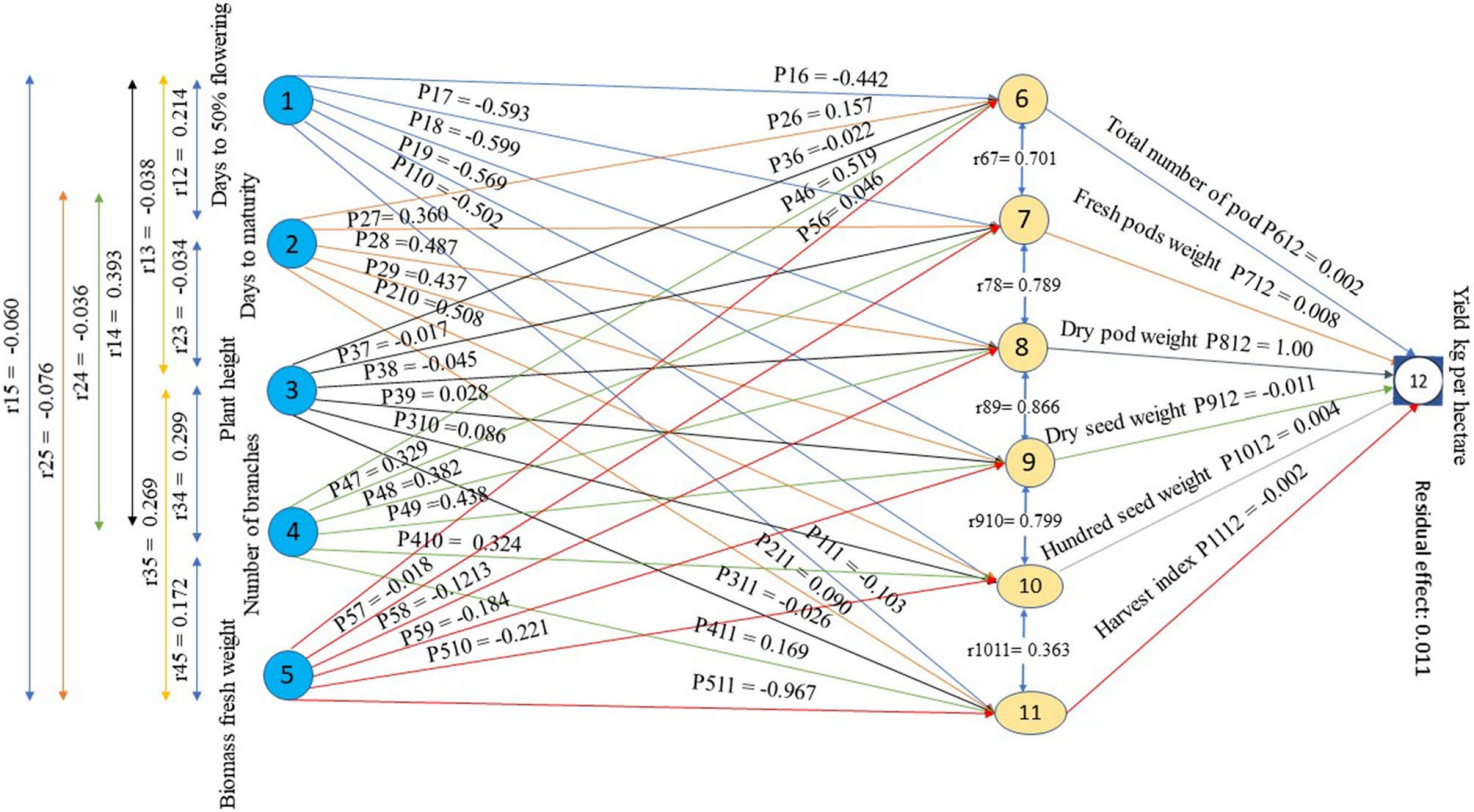
G**e**notypic path coefficient diagram representing cause and effect relationships among quantitative traits and grain yield. Path diagram and coefficients of factors on the influence of first order on the second-order components and the latter on yield kg per hectare of *V. subterranea* genotypes*.* Here, P_*ij*_ is the direct effects and r_*ij*_ are the correlation coefficients. The single arrowed lines in the path diagram reflect direct effect, whereas the double arrowed lines reflect mutual connection. *Note*: 1 = Days to 50% flowering (D50%F); 2 = Days to maturity (DTM); 3 = Plant height (PH); 4 = Number of branches per plant (NB); 5 = Biomass fresh weight (BFW); 6 = Total number of pods per plant (TNP); 7 = Fresh pod weight per plant (FPW); 8 = Dry pod weight per plant (DPW); 9 = Dry seed weight per plant (DSW); 10 = Hundred seed weight (HSW); 11 = Harvest index (HI); 12 = Yield kg/hectare (YLD).

The direction and magnitude of the correlation between yield and yield components is important for determining the important characteristics that may be implemented in the breeding programme as a crop enhancement approach, depending on selective breeding. A simple correlation metric does not accurately capture the characteristics' contribution to pod yield. Path coefficient analysis, on the other hand, is the most commonly used method for studying the interaction of various parameters with pod yield. As displayed in Fig. 1 and Table 8, yield is considered as an artefact of all contributing parameters including days to 50% flowering, days to maturity, plant height, number of branches, biomass fresh weight, total number of pods, fresh pod weight, dry pod weight, hundred seed weight, and harvest index. The correlation coefficients of these contributing traits with yield are categorized as direct and indirect effects (Fig. 1). Wright^30^ distinguishes between direct and indirect effects by assigning correlations for a more precise evaluation of the cause-and-effect relationship. The current study showed a significant correlation between several vegetative, yield, and yield contributing components. Because of their interrelation, these variables have direct and indirect effects on the pod yield and its contributing traits. Consequently, Misangu et al.^31^, Oyiga and Uguru^42^; Ofori^47^, in Bambara groundnut, Usman et al.^19^ in chili, and Oladosu et al.^32^ in rice employ path coefficient analysis effectively to investigate the direct and indirect correlations between component characteristics via the partitioning of correlation coefficients. According to the results of our path analysis, the dry pod weight had the greatest direct effect on yield kg per hectare (1.000), followed by the fresh pod weight (0.0456) and the hundred seed weight (0.0357). Fresh pod weight had the highest indirect influence on yield kg per hectare (0.7890), followed by the total number of pods per plant (0.6358). These characteristics, however, can be used to develop an ideally effective selection index for improving the yield of Bambara groundnut. A similar trend of classification for path coefficient like as very high > 1; 0.3–1 for high; 0.2–0.29 for moderate; 0.1- 0.19 for low; 0.00–0.09 for negligible was noted by Lenka and Mishra^55^ in rice. In reality, a residual effect of 0.011 (Fig. 1) reveals that the causative features explained about 98.90% of the variability in pod yield, leaving 1.10% of the variability unexplored. These findings are corresponded with the result of Aman et al.^56^ who reported 79.6% variability explained by tested traits and leaving 20.4% (residual effect = 0.204) was unexplained when studied in Maize. In our observation, the traits such as days to 50% flowering, plant height, biomass fresh weight, fresh and dry pods weight, hundred seeds weight, pods number, and harvest index showed a positive direct effect to yield. As a result, these traits are considered as the key contributors to overall yield. Makanda et al.^44^ observed similar trends of positive direct contribution. As a result, direct selection based on these traits should be utilized to enhance Bambara groundnut pod yield^42^. This is due to the masking effect of a positive indirect effect of the corresponding traits acknowledged by Oyiga and Uguru^42^, the features with a negative direct effect were days to maturity, dry seed weight, and number of branches per plant. The total number of pods and fresh pods per plant had the highest indirect effect which is consistent with the findings of Misangu et al.^31^, Oyiga and Uguru^42^ in Bambara groundnut.

**Table 8:** The direct (diagonal) and indirect effects of 11 characteristics on pod yield in 30 V*. subterranea* genotypes.

Trait	D50F	DTM	PH	NB	BFW	TNP	FPW	DPW	DSW	HSW	HI
D50F	**0.0209**	0.0045	− 0.0008	0.0082	0.0013	− 0.0042	− 0.0081	− 0.0075	− 0.0066	− 0.0059	− 0.0016
DTM	− 0.0046	− **0.0216**	0.0007	0.0008	0.0017	− 0.0009	− 0.0048	− 0.0079	− 0.0068	− 0.0087	− 0.0030
PH	− 0.0001	− 0.0001	**0.0029**	0.0009	0.0008	0.0005	0.0003	0.0001	0.0003	0.0004	− 0.0007
NB	− 0.0009	0.0001	− 0.0007	− **0.0024**	− 0.0004	− 0.0008	− 0.0002	− 0.0002	− 0.0004	− 0.0002	0.0001
BFW	0.0019	− 0.0025	0.0087	0.0055	**0.0322**	0.0029	− 0.0010	− 0.0045	− 0.0054	− 0.0068	− 0.0308
TNP	0.0061	− 0.0012	− 0.0048	− 0.0104	− 0.0028	− **0.0306**	− 0.0215	− 0.0195	− 0.0174	− 0.0156	− 0.0021
FPW	− 0.0177	0.0102	0.0039	0.0034	− 0.0014	0.0320	**0.0456**	0.0360	0.0345	0.0300	0.0085
DPW	− 0.3603	0.3658	0.0420	0.0945	− 0.1413	0.6358	0.7890	**1.0000**	0.8657	0.7540	0.3601
DSW	0.0168	− 0.0167	− 0.0062	− 0.0094	0.0090	− 0.0302	− 0.0403	− 0.0456	− **0.0533**	− 0.0426	− 0.0194
HSW	− 0.0101	0.0144	0.0045	0.0034	− 0.0075	0.0182	0.0234	0.0269	0.0285	**0.0357**	0.0129
HI	− 0.0026	0.0048	− 0.0083	− 0.0017	− 0.0336	0.0024	0.0066	0.0126	0.0127	0.0127	**0.0351**
Genotypic correlation with Yld	− 0.35**	0.35**	0.042 ns	0.09 ns	− 0.14 ns	0.62**	0.78**	0.99**	0.85**	0.75**	0.36**

D50%F = days to 50% flowering, DTM = days to maturiity, PH = plant height (cm), NB = number of branches , BFW = biomass fresh weight (g), TNP = total number of pods, FWP = fresh pods weight (g), DPW = dry pods weight (g), DSW = dry seeds weight (g), HSW = hundred seed weight (g), HI = harvest index, and YLD = yield kg/ha.

#### Relationships in two stages

The first-order component included vegetative parameters such as days to 50% flowering, days to maturity, plant height, number of branches, and biomass fresh weight. The yield component characteristics comprised the total number of pods, fresh pod weight, dry pod weight, dry seeds weight, hundred seeds weight, and harvest index, which were the major yield determining factors in Bambara groundnut and were included as a second-order component. Tables 6 and 7 demonstrate how these two elements interact with each other.

##### The effects of a first-order component association on a second-order component

The effects of the first-order component on the total number of pods (Table 9) revealed that only plant height and days to maturity had a negative association, whereas all other components had a positive correlation. The number of branches per plant (0.519) had the strongest correlation with the total number of pods, followed by the number of days to maturity (0.157). The path analysis of the first order component with fresh pods weight revealed a negative direct influence on plant height, days to 50% flowering, and biomass fresh weight, but a positive direct influence on other metrics such as days to maturity (0.360) and branch number (0.329). (Table 9). The inter-relationship of days to 50% flowering, plant height, and biomass fresh weight with dry pod weight had a negative direct effect, but the number of branches (0.382) and days to maturity (0.487) showed a positive direct effect (Table 9). Furthermore, days to maturity had the greatest positive and direct influence (0.487) on dry pod weight. Ofori^49^ found that dry pod weight is significantly and indirectly related to the number of branches per plant. The path analysis for the traits revealed that dry seed weight had a direct negative effect on days to 50% flowering and biomass fresh weight. Days to maturity (0.437), plant height (0.028), and branch number (0.438) all exhibited a positive and direct impact on dry seed weight (Table 9). The characteristic hundred seed weight is a significant factor influencing the ultimate yield of Bambara groundnut^20^. In our study, the day to maturity had the most direct positive effect on the hundred seed weight (0.508), followed by the number of branches (0.324). However, biomass fresh weight and day to 50% flowering had a direct negative effect on hundred seed weight (Table 9). Days to maturity, plant height, and biomass fresh weight all had a negative direct association with harvest index, however days to maturity (0.090) and number of branches (0.169) had a positive direct contribution to harvest index (Table 9). We found that the total number of pods per plant had a significant direct effect on on pod yield. Misangu et al.^33^ discovered that plant height had a direct positive association with the number of pods and a strong positive relationship with yield. According to Karikari and Tabone^49^, wider leaves and longer petiole length enhanced canopy and plant height, respectively, which boosted pod yield in Bambara groundnut. According to Misangu et al.^31^, the expansion of a larger canopy encourages the avoidance of moisture loss from the soil, resulting in adequate moisture in the soil to sustain plant development and enhance metabolic rate, which improves crop yield. In Bambara groundnut, pod formation is a significant yield contributing characteristic, and the current study discovered that it has a positive effect on pod yield. Misangu et al.^31^ and Wigglesworth^50^ identified a positive relationship between podding and seed yield in Bambara groundnut. The employment of various genotypes and environments may have led to the variable response, and a number of factors have been identified as having a negative influence among vegetative traits, yield, and its attributed components^31^. Growing the crop under stress-free conditions resulted in a considerable reduction in inter and intra plant competition, resulting in a reduction in yield losses via component compensation^50^.

**Table 9:** First order and second-order component relationships.

Components	Traits	D50F	DTM	PH	NB	BFW
Total number of pods (TNP)	D50F	− **0.4423**	− 0.0947	0.0166	− 0.1739	− 0.0266
	DTM	0.0336	**0.1571**	− 0.0054	− 0.0057	− 0.0120
	PH	0.0009	0.0008	− **0.0227**	− 0.0068	− 0.0061
	NB	0.2041	− 0.0188	0.1550	**0.5191**	0.0893
	BFW	0.0028	− 0.0035	0.0124	0.0079	**0.0460**
	TNP	− 0.2000	0.0408	0.1559	0.340**	0.0905
Fresh pods weight (FPW)	D50F	− **0.5937**	− 0.1272	0.0223	− 0.2334	− 0.0357
	DTM	0.0772	**0.3605**	− 0.0123	− 0.0130	− 0.0275
	PH	0.0006	0.0006	− **0.0171**	− 0.0051	− 0.0046
	NB	0.1296	− 0.0119	0.0984	**0.3296**	0.0567
	BFW	− 0.0011	0.0014	− 0.0050	− 0.0032	− **0.0187**
	FPW	− 0.3874	0.223*	0.0863	0.0749	− 0.0298
Dry pods weight (DPW)	D50F	− **0.5992**	− 0.1284	0.0225	− 0.2356	− 0.0361
	DTM	0.1043	**0.4872**	− 0.0166	− 0.0176	− 0.0372
	PH	0.0017	0.0015	− **0.0454**	− 0.0135	− 0.0122
	NB	0.1502	− 0.0138	0.1141	**0.3821**	0.0657
	BFW	− 0.0073	0.0093	− 0.0327	− 0.0209	− **0.1215**
	DPW	− 0.3503	0.3558	0.0420	0.0945	− 0.1412
Dry seeds weight (DSW)	D50F	− **0.5690**	− 0.1219	0.0214	− 0.2237	− 0.0343
	DTM	0.0938	**0.4378**	− 0.0149	− 0.0158	− 0.0334
	PH	− 0.0011	− 0.0010	**0.0281**	0.0084	0.0076
	NB	0.1723	− 0.0159	0.1309	**0.4384**	0.0754
	BFW	− 0.0111	0.0141	− 0.0495	− 0.0317	− **0.1841**
	DSW	− 0.3150	0.3131	0.1159	0.1756	− 0.1688
Hundred seeds weight (HSW)	D50F	− **0.5025**	− 0.1076	0.0189	− 0.1975	− 0.0303
	DTM	0.1090	**0.5088**	− 0.0174	− 0.0184	− 0.0389
	PH	− 0.0033	− 0.0030	**0.0869**	0.0259	0.0234
	NB	0.1274	− 0.0117	0.0968	**0.3242**	0.0558
	BFW	− 0.0133	0.0169	− 0.0596	− 0.0381	− **0.2216**
	HSW	− 0.2827	0.4034	0.1256	0.0960	− 0.2116
Harvest index (HI)	D50F	− **0.1036**	− 0.0222	0.0039	− 0.0407	− 0.0062
	DTM	0.0193	**0.0902**	− 0.0031	− 0.0033	− 0.0069
	PH	0.0010	0.0009	− **0.0267**	− 0.0080	− 0.0072
	NB	0.0667	− 0.0061	0.0507	**0.1698**	0.0292
	BFW	− 0.0582	0.0739	− 0.2602	− 0.1663	− **0.9671**
	HI	− 0.0748	0.1367	− 0.2353	− 0.0485	− 0.9582

D50%F = days to 50% flowering, DTM = days to maturiity, PH = plant height (cm), NB = number of branches, BFW = biomass fresh weight (g).

##### The effects of a 2nd-order component association on pod yield

The influence of the second-order component on yield is depicted in Table 10. According to the path analysis, dry pod weight (1.00) had the most positive direct effect on seed yield. The second-highest positive and direct contributing attribute was fresh pods weight (0.008), followed by hundred seeds weight (0.004) and the total number of pods (0.002). With the exception of dry pod weight, most of the traits had a poor relationship with yield. In contrast, the harvest index and dry seed weight had a direct negative effect on pod yield. Misangu et al.^31^, Oyiga and Uguru^42^, Maunde et al.^43^, and Makanda et al.^47^ discovered comparable results in the previous study on Bambara groundnut. Nawab et al.^57^ obtained consistent findings in garden pea, while Kumari and Sasidharan^54^ reported equivalent results in groundnut. The researchers identified moderate to strong positive associations between yield and yield component parameters such as total pod number, dry seed weight, and hundred seed weight, whereas our study found a positive but moderate and direct inter-relationship among the variables. According to Wigglesworth^50^, this moderate and negative interaction among components may be owing to competition for ambient resources such as nutrients, light, moisture, geographical coordinates, various meteorological conditions, and the genetic makeup of the landraces. Because of their effect on pod yield, the criteria dry pod weight, total pod number, and hundred seed weight should be prioritized during selection in the Bambara groundnut improvement program.

**Table 10:** Second-order components’ effects on yield.

Trait	TNP	FPW	DPW	DSW	HSW	HI
TNP	**0.002**	0.001	0.001	0.001	0.001	0.000
FPW	0.005	**0.008**	0.006	0.006	0.005	0.001
DPW	0.580	0.789	**1.000**	0.856	0.754	0.360
DSW	− 0.006	− 0.009	− 0.010	− **0.011**	− 0.009	− 0.004
HSW	0.002	0.003	0.003	0.004	**0.004**	0.002
HI	0.040	0.000	− 0.001	− 0.001	− 0.001	− **0.002**
Yld	0.62**	0.79**	0.99**	0.85**	0.75**	0.36**

TNP = total number of pods, FWP = fresh pods weight (g), DPW = dry pods weight (g), DSW = dry seeds weight (g), HSW = hundred seed weight (g), HI = harvest index, and YLD = yield kg/ha.

## Conclusion

In the conclusion, the investigation suggested that dry pods weight per plant was the most key attribute among the tested yield components traits in Bambara groundnut due to its strong and positive correlation as well as a high direct impact to yield kg per hectare. This one was accompanied by that of the number of pods per plant, hundred seed weight, and fresh pods weight which also displayed strong and positive correlation as well as a high direct effect to yield kg per hectare. This implies that in the case of direct selection for high yielding genotypes above mentioned traits should also be emphasized selection. The indirect effect via dry pods weight is also important for the significant correlations with the number of total pods, fresh pods weight, dry seed weight, harvest index, and hundred seed weight. The indirect effects were usually low indicating that positive correlations among the traits were largely due to the direct effects. The dried pod weight had the most direct beneficial influence on yield, followed by the fresh pod weight. Following the total number of pods and dry pods weight, fresh pod weight had the highest indirect effect on yield per hectare. As a result, to maximize the production of *Vigna subterranea*, the variables total number of pods, fresh and dry pods weight, and hundred seed weight must be prioritized.

## Supplementary Information

Supplementary Information.

## Data Availability

All data are provided in the manuscript's text body. We also certify that a voucher specimen of the described species has been placed in a publicly accessible herbarium and GenBank, ITAFoS, Universiti Putra Malaysia (UPM). The deposition number is Bambara groundnut (*Vigna subterranea*)/ITAFoS/UPM/S5-2021.

## References

[1] Ntundu WH, Bach IC, Christiansen JL, Andersen SB (2004). Analysis of genetic diversity in bambara groundnut [*Vigna subterranea* (L.) Verdc] landraces using amplified fragment length polymorphism (AFLP) markers. Afr. J. Biotechnol..

[2] Khan MMH, Rafii MY, Ramlee SI, Jusoh M, Al Mamun M, Halidu J (2021). DNA fingerprinting, fixation-index (Fst), and admixture mapping of selected Bambara groundnut (*Vigna subterranea* [L.] Verdc.) accessions using ISSR markers system. Sci. Rep..

[3] Khan MMH, Rafii MY, Ramlee SI, Jusoh M, Al-Mamun M (2021). Bambara groundnut (*Vigna**subterranea* L. Verdc): A crop for the new millennium, its genetic diversity, and improvements to mitigate future food and nutritional challenges. Sustainability.

[4] Linnemann AR, Westphal E, Wessel M (1995). Photoperiod regulation of development and growth in bambara groundnut (*Vigna**subterranea*). Field Crop Res.

[5] Sesay A, Magagula CN, Mansuetus AB (2008). Influence of sowing date and environmental factors on the development and yield of bambara groundnut (*Vigna**subterranea*) landraces in a sub-tropical region. Exp. Agric..

[6] Collinson ST, Berchie J, Azam-Ali SN (1999). The effect of soil moisture on light interception and the conversion coefficient for three landraces of bambara groundnut (*Vigna**subterranea*). J. Agric. Sci..

[7] Mwale SS, Azam-Ali SN, Massawe FJ (2007). Growth and development of bambara groundnut (*Vigna**subterranea*) in response to soil moisture: 1. Dry matter and yield. Eur. J. Agron..

[8] Massawe FJ, Azam-Ali SN, Roberts JA (2003). Impact of temperature on leaf development in bambara groundnut landraces. Crop Sci..

[9] Collinson ST, Azam-Ali SN, Chavula KM, Hodson DA (1996). Growth, development and yield of bambara groundnut (*Vigna**subterranea*) in response to soil moisture. J. Agric. Sci..

[10] Khan MMH, Rafii MY, Ramlee SI, Jusoh M, Al Mamun M (2021). AMMI and GGE biplot analysis for yield performance and stability assessment of selected Bambara groundnut (*Vigna**subterranea* L. Verdc.) genotypes under the multi-environmental trails (METs). Sci. Rep..

[11] Khan, M. M. H., Rafii, M. Y., Ramlee, S. I., Jusoh, M., & Mamun, A. Genetic variability, heritability, and clustering pattern exploration of bambara groundnut (*Vigna subterranea* L. Verdc) Accessions for the Perfection of Yield and Yield-Related Traits. *BioMed Res. Int.*, *2020*. (2020). 10.1155/2020/2195797 PMC7769641 33415143

[12] Khan MMH, Rafii MY, Ramlee SI, Jusoh M, Al Mamun M (2021). Genetic analysis and selection of Bambara groundnut (*Vigna**subterranea* [L.] Verdc) landraces for high yield revealed by qualitative and quantitative traits. Sci. Rep..

[13] Obidiebube EA, Eruotor PG, Akparaobi SO, Okolie H, Obasi CC (2020). Evaluation of Bambara groundnut (*Vigna**Subterranea* (L) Verdc.) varieties for adaptation to rainforest agroecological zone of delta state, Nigeria. Evaluation.

[14] Molosiwa, O. O. *Genetic diversity and population structure analysis of bambara groundnuts (Vigna subterranea (L.) Verdc.) landraces using morpho-agronomic characters and SSR markers* (Doctoral dissertation, University of Nottingham). (2012).

[15] Yaghotipoor A, Farshadfar E (2007). Non-parametric estimation and component analysis of phenotypic stability in chickpea (*Cicer**arietinum* L.). PJBS.

[16] Puttha R, Jogloy S, Wongkaew S, Sanitchon J, Kesmala T, Patanothai A (2008). Heritability, phenotypic and genotypic correlation of peanut bud necrosis virus resistance and agronomic traits in peanut. Asian J. Plant Sci..

[17] Painawadee M, Jogloy S, Kesmala T, Akkasaeng C, Patanothai A (2009). Heritability and correlation of drought resistance traits and agronomic traits in peanut (*Arachis hypogaea* L.). Asian J. Plant Sci..

[18] Duran LA, Blair MW, Giraldo MC, Macchiavelli R, Prophète E, Nin JC, Beaver JS (2005). Morphological and molecular characterization of common bean landraces and cultivars from the Caribbean. Crop Sci..

[19] Usman MG, Rafii MY, Martini MY, Oladosu Y, Kashiani P (2017). Genotypic character relationship and phenotypic path coefficient analysis in chili pepper genotypes grown under tropical condition. J. Sci. Food Agric..

[20] Masindeni, D. R. *Evaluation of bambara groundnut (Vigna subterranea) for yield stability and yield related characteristics* (Doctoral dissertation, University of the Free State). (2006).

[21] Anwar J, Ali MA, Hussain M, Sabir W, Khan MA, Zulkiffal M, Abdullah M (2009). Assessment of yield criteria in bread wheat through correlation and path analysis. J. Anim. Plant Sci..

[22] Naserian B, Asadi AA, Rahimi M, Ardakani MR (2007). Evaluation of wheat cultivar mutants for morphological and yield traits and comparing of yield components under irrigated and rainfed conditions. Asian J. Plant Sci..

[23] Basalma D (2008). The correlation and path analysis of yield and yield components of different winter rapeseed (*Brassica **napus* ssp. oleifera L.) cultivars. Res. J. Agric. Biol. Sci..

[24] Manggoel W, Uguru MI, Ndam ON, Dasbak MA (2012). Genetic variability, correlation and path coefficient analysis of some yield components of ten cowpea [*Vigna unguiculata* (L.) Walp] accessions. J. Plant Breed. Crop Sci..

[25] Falconer DS, Mackay TF, Frankham R (1996). Introduction to quantitative genetics (4th edn). Trends Genetics.

[26] Maniee M, Kahrizi D, Mohammadi R (2009). Genetic variability of some morpho-physiological traits in durum wheat (*Triticum turgidum* var. durum). J. Appl. Sci..

[27] Lestari SADAD, Melati M, Purnamawati H (2015). Penentuan dosis optimum pemupukan n, p, dan k pada tanaman kacang Bogor [*Vigna subterranea* (L) Verdcourt]. Indon. J. Agron..

[28] IPGRI, IITA, BAMNET. (2000). BAMNET. Descriptors for Bambara Groundnut (Vigna subterranea). International Plant Genetic Resources Institute, Rome, Italy; International Institute of Tropical Agriculture, Ibadan, Nigeria. *The International Bambara Groundnut Network, Germany*, *57*.

[29] Kashiani P, Saleh G (2010). Estimation of genetic correlations on sweet corn inbred lines using SAS mixed model. Am. J. Agric. Biol. Sci..

[30] Wright S (1921). Correlation and causation. J. Agric. Res..

[31] Misangu RN, Azmio A, Reuben SOWM, Kusolwa PM, Mulungu LS (2007). Path coefficient analysis among components of yield in bambara groundnut (*Vigna subterranea* L. Verdc) landraces under screen house conditions. J. Agron..

[32] Oladosu Y, Rafii MY, Magaji U, Abdullah N, Miah G, Chukwu SC, Hussin G, Ramli A, Kareem I (2018). Genotypic and phenotypic relationship among yield components in rice under tropical conditions. BioMed Res. Int..

[33] Oladosu Y, Rafii MY, Abdullah N, Malek MA, Rahim HA, Hussin G, Ismail MR, Latif MA, Kareem I (2015). Genetic variability and diversity of mutant rice revealed by quantitative traits and molecular markers. Agrociencia.

[34] Suneetha, N. *Morphological and Rapd Based Gene Diversity Studies Among Released Cultivars and Pre-Release Cultures of Groundnut (Arachis Hypogaea L.)* (Doctoral dissertation, Acharya Ng Ranga Agricultural University, Rajendranagar, Hyderabad). (2007)

[35] Pranesh H, Nandini R, Chandra K, Rangaiah S, Nagaraju N (2017). Character association and path analysis of yield and yield components in m3 generation of bambara groundnut (*Vigna** subterranean* (L.) verdc) treated with ethyl methane sulphonate (EMS). Int. J. Pure Appl. Biosci..

[36] Shegro A, Van Rensburg WJ, Adebola PO (2013). Assessment of genetic variability in bambara groundnut (*Vigna subterrenea* L. Verdc) using morphological quantitative traits. Acad. J. Agric. Res..

[37] Jonah PM, Aliyu B, Jibung GG, Abimiku OE (2013). Phenotypic and genotypic variance and heritability estimates in bambara groundnut (*Vigna subterranea* [L] Verdc) in Mubi, Adamawa State, Nigeria. Int J. IT Eng. Appl. Sci. Res..

[38] Naik, U. *Correlation and Path Coefficient Analysis Between Seed Yield and Its Component Characters in M4 and M5 Generations of Bambara Groundnut* (Doctoral dissertation, University of Agricultural Sciences, GKVK). (2015).

[39] Mohammed MS, Shimelis HA, Laing MD (2020). Preliminary morphological characterization and evaluation of selected Bambara groundnut [*Vigna subterranea* (L.) Verdc] genotypes for yield and yield related traits. Legume Res. Int. J..

[40] Ambrose, S. F. *Genotype by environment interaction in groundnut genotypes for yield and other agronomic traits in two locations in Ghana* (Doctoral dissertation). (2015).

[41] Ratner B (2009). The correlation coefficient: Its values range between & plus, 1/–1, or do they & quest. J. Target. Meas. Anal. Mark..

[42] Oyiga BC, Uguru MI (2011). Interrelationships among pod and seed yield traits in bambara groundnut (*Vigna subterranea* L. Verdc) in the derived savanna agro-ecology of south-eastern Nigeria under two planting dates. Int. J. Plant Breed..

[43] Maunde SM, Tanimu B, Mahmud M (2015). Correlation and path coefficient analysis of yield characters of bambara (*Vigna subterranea* L. Verdc.). Afr. J. Environ. Sci. Technol..

[44] Makanda I, Tongoona P, Madamba R, Icishahayo D, Derera J (2009). Path coefficient analysis of bambara groundnut pod yield components at four planting dates. Res. J. Agric. Biol. Sci..

[45] Alake CO, Ayo-Vaughan MA, Ariyo JO (2015). Selection criteria for grain yield and stability in bambara groundnut (*Vigna** subterranean* (L) Verdc) landraces. Acta Agric. Scand. Sect. B Soil Plant Sci..

[46] Unigwe AE, Gerrano AS, Adebola P, Pillay M (2016). Morphological variation in selected accessions of Bambara groundnut (*Vigna subterranea* (L.) Verdc) in South Africa. J. Agricult. Sci..

[47] Ofori I (1996). Correlation and path-coefficient analysis of components of seed yield in bambara groundnut (*Vigna subterranea*). Euphytica.

[48] Iqbal, S., Mahmood, T., Ali, M., Anwar, M., & Sarwar, M. Path coefficient analysis in different genotypes of soybean (*Glycine max* (L) Merril). *Pak. J. Biol. Sci. (Pakistan)*. (2003).

[49] Karikari SK, Tabona TT (2004). Constitutive traits and selective indices of Bambara groundnut (*Vigna subterranea* (L) Verdc) landraces for drought tolerance under Botswana conditions. Phys. Chem. Earth A/B/C.

[50] Wigglesworth, D. J. (1996, July). The potential for genetic improvement of bambara groundnut (*Vigna subterranea* L. Verdc.) in Botswana. In *Proceedings of the International Symposium on Bambara groundnut* (pp. 23–25).

[51] Mogale, T. E. *Multi-Location Field Evaluation of Bambara Groundnut (Vigna subterranean (L) Verdc) for Agronomic Performance and Seed Protein* (Doctoral dissertation). (2018).

[52] Evangeline, U. A. *Assessing the morphological variation and characterising the proteins of bambara groundnut (Vigna Subterranea L. Verdc)* (Doctoral dissertation, Vaal University of Technology). (2016).

[53] Jonah PM (2011). Phenotypic and genotypic correlation in bambara groundnut (*Vigna subterranea* (L.) Verdc) in Mubi, Adamawa State, Nigeria. World J. Agric. Sci..

[54] Kumari K, Sasidharan N (2020). Studies on genetic variability, correlation and path coefficient analysis for morphological and yield traits in different Arachis spp. Int. J. Curr. Microbiol. App. Sci.

[55] Lenka D, Mishra B (1973). Path coefficient analysis of yield in rice varieties. Indian J. Agric. Sci.

[56] Aman J, Bantte K, Alamerew S, Sbhatu DB (2020). Correlation and path coefficient analysis of yield and yield components of quality protein maize (*Zea** mays* L.) hybrids at Jimma, western Ethiopia. Int J Agron.

[57] Nawab, N. N., Subhani, G. M., Mahmood, K., Shakil, Q., & Saeed, A. (2008). Genetic variability, correlation and path analysis studies in garden pea (*Pisum sativum* L.). *J. Agric. Res. (Pak.)*.

