# Supplementary material for: Path-coefficient and correlation analysis in Bambara groundnut (Vigna subterranea [L.] Verdc.) accessions over environments
Source: Sci Rep. 2022 Jan 7;12:245. doi: 10.1038/s41598-021-03692-z (PMC8742022; doi:10.1038/s41598-021-03692-z)
Supplement: Supplementary file 1 — Supplementary Information. [file 41598_2021_3692_MOESM1_ESM.pdf]

Supplementary Information

Path-coefficient and Correlation Analysis in Bambara groundnut (*Vigna subterranea* [L.] Verdc.) Accessions Over Environments

Md Mahmudul Hasan Khan[1, 3*], Mohd Y. Rafii[1, 2*], Shairul Izan Ramlee[2], Mashitah Jusoh[2] , Md Al Mamun[1, 4],

[1] Laboratory of Climate-Smart Food Crop Production, Institute of Tropical Agriculture and Food Security (ITAFoS), Universiti Putra Malaysia (UPM), 43400, UPM Serdang, Selangor, Malaysia.

[2]Department of Crop Science, Faculty of Agriculture, Universiti Putra Malaysia (UPM), 43400, UPM Serdang, Selangor, Malaysia.

[3] Bangladesh Agricultural Research Institute (BARI), Gazipur-1701, Bangladesh.

[4]Bangladesh Jute Research Institute (BJRI), Dhaka, Bangladesh.

*Correspondence: M.Y. Rafii; mrafii@upm.edu.my, Tel.: +60-3-97691043, and M.M.H. Khan; mhasan.bari12@gmail.com. Tel.: +60-109187125

Supplementary tables

Table S1: The sets of simultaneous equations which were organized in matrix notation to highlight the relationships between correlations and path coefficients

In these equations, r denotes the phenotypic correlations between variables, P denotes the coefficients (direct effects) of one variable on another, and ri:jPi:j denotes the indirect effects. The following sequential number of each observation is used to identify it.

1 = Days to 50% flowering (D50%F)
2 = Days to maturity (DTM).
3 = Plant height (PH)
4 = Number of branches per plant (NB)
5 = Biomass fresh weight (BFW)
6 = Total number of pods per plant (TNP)
7 = Fresh pod weight per plant (FPW)
8 = Dry seed weight per plant (DSW)
9 = Hundred seed weight (HSW)
10 = Harvest index (HI)
11 = Dry pod weight per plant (DPW)

Effects of vegetative and yield component traits on dry pod weight (DPW)

$r_{1:11} = P_{1:11} + r_{1:2}P_{2:11} + r_{1:3}P_{3:211} + r_{1:4}P_{4:11} + r_{1:5}P_{5:11} + r_{1:6}P_{6:11} + r_{1:7}P_{7:11} + r_{1:8}P_{8:11} + r_{1:9}P_{911} + r_{1:10}P_{10:11}$

$r_{2:11} = r_{2:1}P_{1:11} + P_{2:11} + r_{2:3}P_{3:11} + r_{2:4}P_{4:11} + r_{2:5}P_{5:11} + r_{2:6}P_{6:11} + r_{2:7}P_{7:11} + r_{2:8}P_{8:11} + r_{2:9}P_{9:11} + r_{2:10}P_{10:11}$

$r_{3:11} = r_{3:1}P_{1:11} + r_{3:2}P_{2:11} + P_{3:11} + r_{3:4}P_{4:11} + r_{3:5}P_{5:11} + r_{3:6}P_{6:11} + r_{3:7}P_{7:11} + r_{3:8}P_{8:11} + r_{3:9}P_{9:11} + r_{3:10}P_{10:11}$

$r_{4:11} = r_{4:1}P_{1:11} + r_{4:2}P_{2:11} + r_{4:3}P_{3:11} + P_{4:11} + r_{4:5}P_{5:11} + r_{4:6}P_{6:11} + r_{4:7}P_{7:11} + r_{4:8}P_{8:11} + r_{4:9}P_{9:11} + r_{4:10}P_{10:11}$

$r_{5:11} = r_{5:1}P_{1:11} + r_{5:2}P_{2:11} + r_{5:3}P_{3:11} + r_{5:4}P_{4:11} + P_{5:11} + r_{5:6}P_{6:11} + r_{5:7}P_{7:11} + r_{5:8}P_{8:11} + r_{5:9}P_{9:11} + r_{5:10}P_{10:11}$

$r_{6:11} = r_{6:1}P_{1:11} + r_{6:2}P_{2:11} + r_{6:3}P_{3:11} + r_{6:4}P_{4:11} + r_{6:5}P_{5:11} + P_{6:11} + r_{6:7}P_{7:11} + r_{6:8}P_{8:11} + r_{6:9}P_{9:11} + r_{6:10}P_{10:11}$

$r_{7:11} = r_{7:1}P_{1:11} + r_{7:2}P_{2:11} + r_{7:3}P_{3:11} + r_{7:4}P_{4:11} + r_{7:5}P_{5:11} + r_{7:6}P_{6:11} + P_{7:11} + r_{78}P_{8:11} + r_{7:9}P_{9:11} + r_{7:10}P_{10:11}$

$r_{8:11} = r_{8:1}P_{1:11} + r_{8:2}P_{2:11} + r_{8:3}P_{3:11} + r_{8:4}P_{4:11} + r_{8:5}P_{5:11} + r_{8:6}P_{6:11} + r_{8:7}P_{7:11} + P_{8:11} + r_{8:9}P_{9:11} + r_{8:10}P_{10:11}$

$r_{9:11} = r_{9:1}P_{1:11} + r_{9:2}P_{2:11} + r_{9:3}P_{3:11} + r_{9:4}P_{4:11} + r_{9:5}P_{5:11} + r_{9:6}P_{6:11} + r_{9:7}P_{7:11} + r_{9:8}P_{8:11} + P_{9:11} + r_{9:10}P_{10:11}$

$r_{10:11} = r_{10:1}P_{1:11} + r_{10:2}P_{2:11} + r_{10:3}P_{3:11} + r_{10:4}P_{4:11} + r_{10:5}P_{5:11} + r_{10:6}P_{6:11} + r_{10:7}P_{7:11} + r_{10:8}P_{8:11} + r_{10:9}P_{9:11} + P_{10:11}$

The equation matrix of the first-order components effects on total number of pods per plant (TNP), fresh pod weight per plant (FPW), dry pod weight per plant (DPW), dry seed weight per

plant (DSW), hundred seed weight (HSW), and harvest index (HI) are as follows:

Total number of pods per plant (TNP)

$r_{1:6} = P_{1:6} + r_{1:2}P_{2:6} + r_{1:3}P_{3:6} + r_{1:4}P_{4:6} + r_{1:5}P_{5:6}$

$r_{2:6} = r_{2:1}P_{1:6} + P_{2:6} + r_{2:3}P_{3:6} + r_{2:4}P_{4:6} + r_{2:5}P_{5:6}$

$r_{3:6} = r_{3:1}P_{1:6} + r_{3:2}P_{2:6} + P_{3:6} + r_{3:4}P_{4:6} + r_{3:5}P_{5:6}$

$r_{4:6} = r_{4:1}P_{1:6} + r_{4:2}P_{2:6} + r_{4:3}P_{3:6} + P_{4:6} + r_{4:5}P_{5:6}$

$r_{5:6} = r_{5:1}P_{1:6} + r_{5:2}P_{2:6} + r_{5:3}P_{3:6} + r_{5:4}P_{4:6} + P_{5:6}$.

Fresh pod weight per plant (FPW)

$r_{1:7} = P_{1:7} + r_{1:2}P_{2:7} + r_{1:3}P_{3:7} + r_{1:4}P_{4:7} + r_{1:5}P_{5:7}$

$r_{2:7} = r_{2:1}P_{1:7} + P_{2:7} + r_{2:3}P_{3:7} + r_{2:4}P_{4:7} + r_{2:5}P_{5:7}$

$r_{3:7} = r_{3:1}P_{1:7} + r_{3:2}P_{2:7} + P_{3:7} + r_{3:4}P_{4:7} + r_{3:5}P_{5:7}$

$r_{4:7} = r_{4:1}P_{1:7} + r_{4:2}P_{2:7} + r_{4:3}P_{3:7} + P_{4:7} + r_{4:5}P_{5:7}$

$r_{5:7} = r_{5:1}P_{1:7} + r_{5:2}P_{2:7} + r_{5:3}P_{3:7} + r_{5:4}P_{4:7} + P_{5:7}$.

Dry seed weight per plant (DSW)

$r_{1:8} = P_{1:8} + r_{1:2}P_{2:8} + r_{1:3}P_{3:8} + r_{1:4}P_{4:8} + r_{1:5}P_{5:8}$

$r_{2:8} = r_{2:1}P_{1:8} + P_{2:8} + r_{2:3}P_{3:8} + r_{2:4}P_{4:8} + r_{2:5}P_{5:8}$

$r_{3:8} = r_{3:1}P_{1:8} + r_{3:2}P_{2:8} + P_{3:8} + r_{3:4}P_{4:8} + r_{3:5}P_{5:8}$

$r_{4:8} = r_{4:1}P_{1:8} + r_{4:2}P_{2:8} + r_{4:3}P_{3:8} + P_{4:8} + r_{4:5}P_{5:8}$

$r_{5:8} = r_{5:1}P_{1:8} + r_{5:2}P_{2:8} + r_{5:3}P_{3:8} + r_{5:4}P_{4:8} + P_{5:8}$.

Hundred seed weight (HSW)

$r_{1:9} = P_{1:9} + r_{1:2}P_{2:9} + r_{1:3}P_{3:9} + r_{1:4}P_{4:9} + r_{1:5}P_{5:9}$

$r_{2:9} = r_{2:1}P_{1:9} + P_{2:9} + r_{2:3}P_{3:9} + r_{2:4}P_{4:9} + r_{2:5}P_{5:9}$

$r_{3:9} = r_{3:1}P_{1:9} + r_{3:2}P_{2:9} + P_{3:9} + r_{3:4}P_{4:9} + r_{3:5}P_{5:9}$

$r_{4:9} = r_{4:1}P_{1:9} + r_{4:2}P_{2:9} + r_{4:3}P_{3:9} + P_{4:9} + r_{4:5}P_{5:9}$

$r_{5:9} = r_{5:1}P_{1:9} + r_{5:2}P_{2:9} + r_{5:3}P_{3:9} + r_{5:4}P_{4:9} + P_{5:9}$.

Harvest index (HI)

$r_{1:10} = P_{1:10} + r_{1:2}P_{2:10} + r_{1:3}P_{3:10} + r_{1:4}P_{4:10} + r_{1:5}P_{5:10}$

$r_{2:10} = r_{2:1}P_{1:10} + P_{2:10} + r_{2:3}P_{3:10} + r_{2:4}P_{4:10} + r_{2:5}P_{5:10}$

$r_{3:10} = r_{3:1}P_{1:10} + r_{3:2}P_{2:10} + P_{3:10} + r_{3:4}P_{4:10} + r_{3:5}P_{5:10}$

$r_{4:10} = r_{4:1}P_{1:10} + r_{4:2}P_{2:10} + r_{4:3}P_{3:10} + P_{4:10} + r_{4:5}P_{5:10}$

$r_{5:10} = r_{5:1}P_{1:10} + r_{5:2}P_{2:10} + r_{5:3}P_{3:10} + r_{5:4}P_{4:10} + P_{5:10}$.

Dry pod weight per plant (DPW)

$r_{1:11} = P_{1:11} + r_{1:2}P_{2:11} + r_{1:3}P_{3:11} + r_{1:4}P_{4:11} + r_{1:5}P_{5:11}$

$r_{2:11} = r_{2:1}P_{1:11} + P_{2:11} + r_{2:3}P_{3:11} + r_{2:4}P_{4:11} + r_{2:5}P_{5:11}$

$r_{3:11} = r_{3:1}P_{1:11} + r_{3:2}P_{2:11} + P_{3:11} + r_{3:4}P_{4:11} + r_{3:5}P_{5:11}$

$r_{4:11} = r_{4:1}P_{1:11} + r_{4:2}P_{2:11} + r_{4:3}P_{3:11} + P_{4:11} + r_{4:5}P_{5:11}$

$r_{5:11} = r_{5:1}P_{1:11} + r_{5:2}P_{2:11} + r_{5:3}P_{3:11} + r_{5:4}P_{4:11} + P_{5:11}$.
